# Supplementary material for: Superior colliculus modulates cortical coding of somatosensory information
Source: Nat Commun. 2020 Apr 3;11:1693. doi: 10.1038/s41467-020-15443-1 (PMC7125203; doi:10.1038/s41467-020-15443-1)
Supplement: Supplementary file 1 — Supplementary Information [file 41467_2020_15443_MOESM1_ESM.pdf]

Superior colliculus modulates cortical coding of somatosensory information

Gharaei et al.

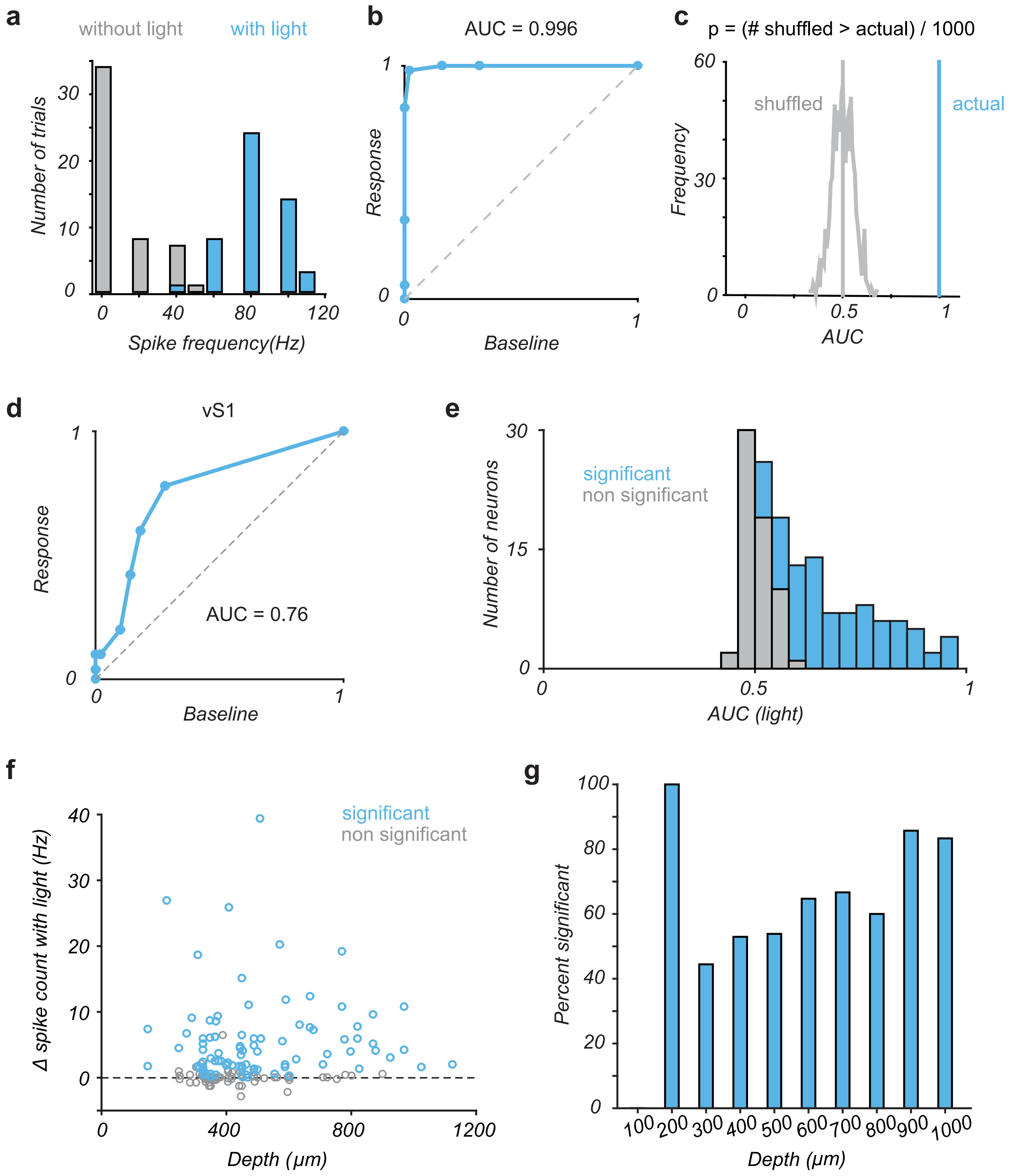

Supplementary Fig. 1: ROC analysis and impact of SC activation across depth.
a. Trial-by-trial spike count distributions of an example SC neuron (same neuron as Fig. 1c) for optogenetic stimulation (blue) and the baseline (grey). b. ROC curve of the same example neuron during light stimulation. The dashed line shows what is expected by chance. For this neuron, the area under the ROC curve (AUC) is 0.996. c. The distribution of 1000 bootstrapped (i.e. shuffled) AUCvalues (grey) and the observed AUC value for this example neuron (blue line). d. ROC curve for an example vS1 neuron during optogenetic stimulation of SC. The dashed line shows what is expected by chance. For this neuron, the AUC value is 0.76. e. The distribution of AUC values across all neurons (n=149). Grey depicts vS1 neurons where there was no significant change in spiking and blue (n=87) shows vS1 neurons with a significant increase in spiking in response to SC stimulation. f. Change in spiking during optogenetic activation of SC compared to baseline versus recording depth in vS1 (n=149). Cells with a significant increases in spiking are shown in blue (ROC analysis). g. Percentage of recorded neurons that responded significantly to light versus recording depth. Source data are provided as a Source Data file.

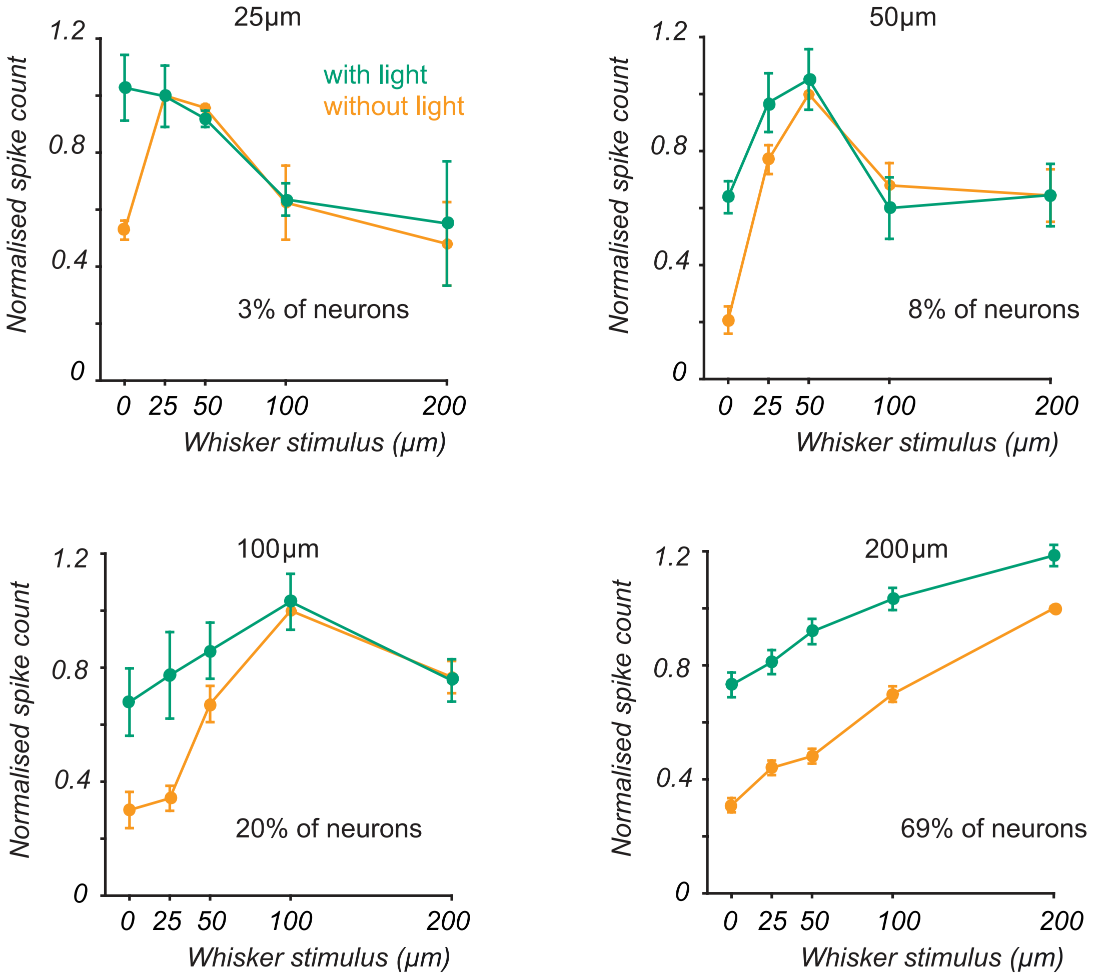

Supplementary Fig. 2: Impact of SC on whisker tuning.
Whisker responsive vS1 neurons that also responded to SC activation (n=101) were divided into 4 groups depending on the whisker stimulus that evoked the maximum response (either 25, 50, 100 or 200 µm). Spiking activity of each group was normalized to the maximum response to whisker stimulation alone. Seventy neurons (69%) had the greatest response to 200 µm whisker deflections (bottom right), 20 neurons (20%) had the greatest response to 100 µm whisker deflections (bottom, left), 8 neurons (8%) had the greatest response to 50 µm whisker defections (top, right) and 3 neurons (3%) had the greatest response to 25 µm defections (top, left). For every group, optogenetic activation of SC only impacted on whisker responses for deflection amplitudes lower than that which evoked the maximum response. Error bars represent SEM. Source data are provided as a Source Data file.

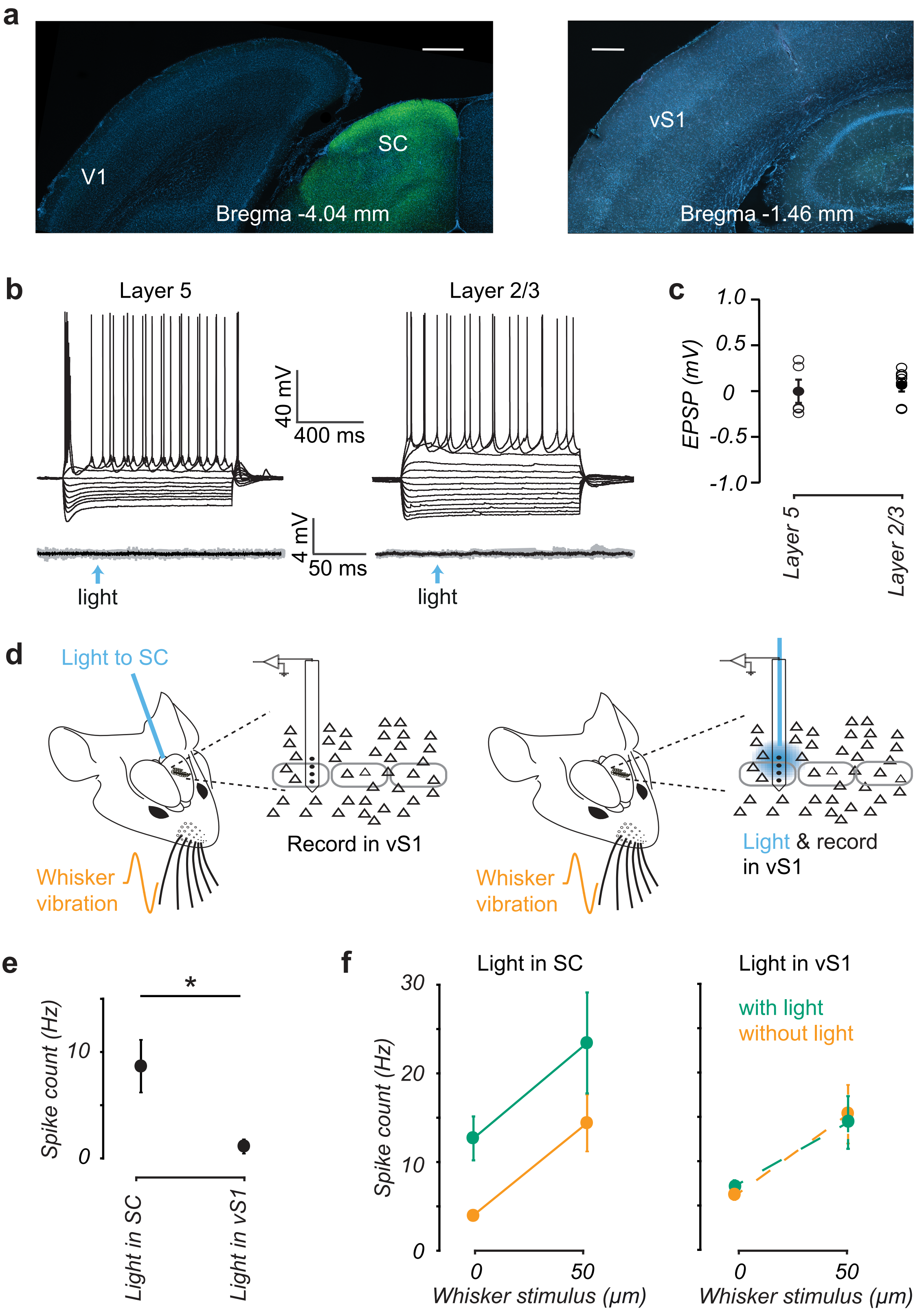

Supplementary Fig. 3: SC modulation of vS1 is not due to retrograde transport of AA1.

a. Left: Coronal section showing expression of AAV1-ChR2-eYFP restricted to SC (green). Scale bar is 500 μm. DAPI in blue. V1: primary visual cortex. Right: Coronal section from the same aniaml shows an absence of expression in vS1. Scale bar is 200 μm. DAPI in blue. b. Top: Response of a layer 5 and a layer 2/3 neuron in vS1 to somatic depolarizing and hyperpolarizing current injections. Bottom: Voltage response of the same neurons to photo-activation at the maximum LED power (2 ms; 5 mW). c. Summary of EPSP amplitudes in layer 5 (n=7) and layer 2/3 (n=5) vS1 neurons during photo-activation at the highest LED power tested (5 mW). d. Schematic of the experimental arrangement. Left: Extracellular recording in vS1 and optogenetic activation of SC with or without whisker vibration. Right: Extracellular recording and optogenetic activation in vS1 with or without whisker vibration. e. Plot of baseline-subtracted responses in vS1 to optogenetic activation of either SC or vS1 (n=12). Asterisk represents p = 0.014 (Two-sided paired t-test). f. Pooled data (n=12 neurons) showing the impact of light (green) on spiking in vS1 neurons during 50 μm whisker movements (orange). Left: Optogenetic activation of SC. Right: Optogenetic activation of vS1. Error bars represent SEM. Source data are provided as a Source Data file.

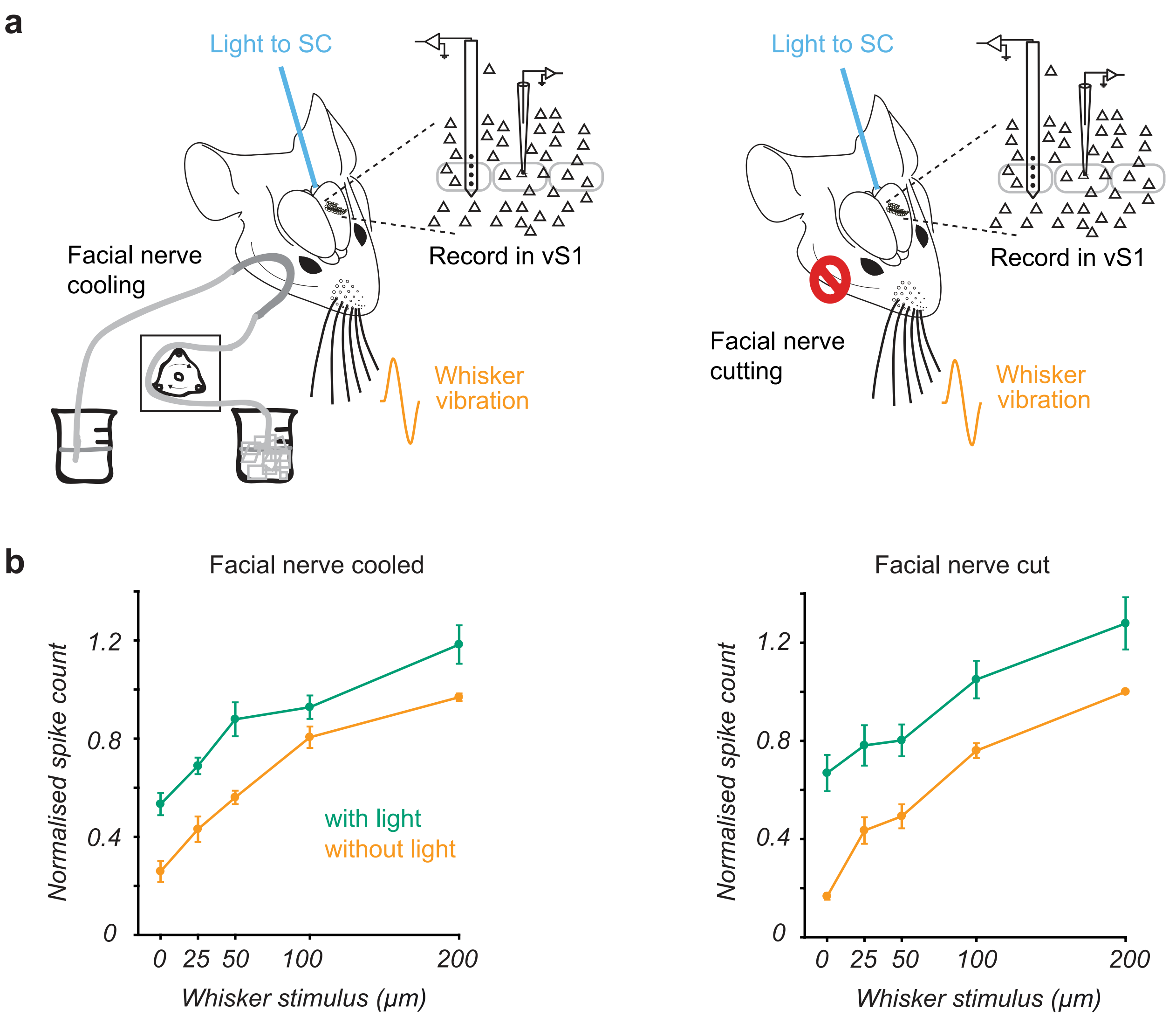

Supplementary Fig. 4: Comparison of the impact of facial nerve block by cooling or cutting.
a. Schematic of the experimental arrangement. The impact of optogenetic activation of SC on vS1 responses was measured before and after facial nerve cooling (left) or cutting (right). b. Pooled data showing the impact of SC activation (green) on the whisker input-output relationship of vS1 neurons (orange) after facial nerve inactivation by cooling (left; n=16) or cutting (right; n=8). The spiking of each neuron was normalized to the maximum response to whisker stimulation alone. Error bars represent SEM. Source data are provided as a Source Data file.

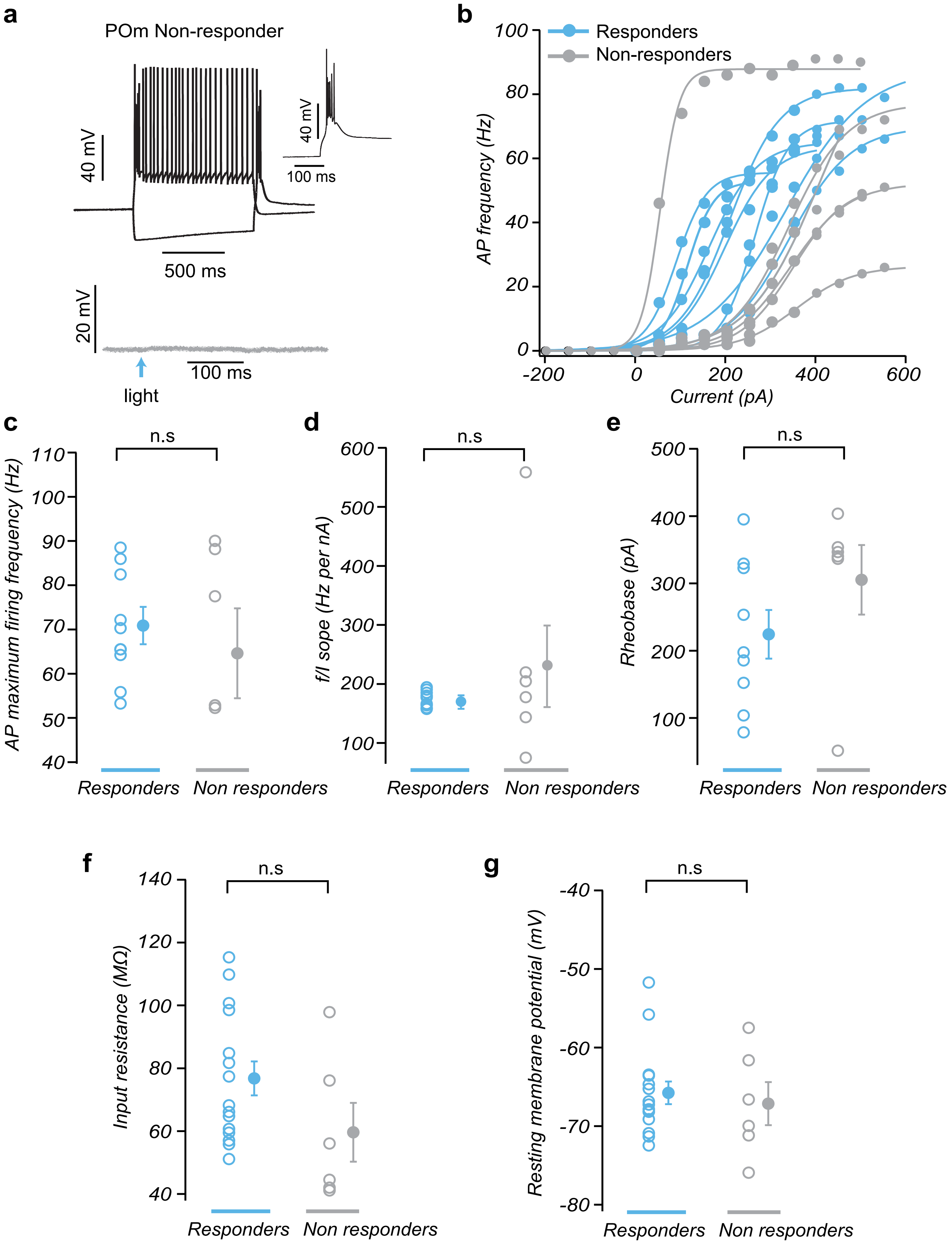

Supplementary Fig. 5: POm neurons that receive input from SC have similar active and passive properties to those that do not.

a. Top: Response of a POm neuron to somatic depolarizing (+300 pA) and hyperpolarizing (-400 pA) current steps. Inset shows the rebound spikes. Bottom: This POm neuron does not receive SC input. b. Plot of action potential (AP) firing frequency versus somatic current (f/I) in POm neurons that receive SC input (“responders”; blue; n=9) and those that do not (“non-responders”; grey; n=6). c. Comparison of maximum AP firing frequency in “responders” (n=9) and “non-responders” (n=6). d. Comparison of f/I slope in “responders” (n=9) and “non-responders” (n=6). The slope was calculated by fitting a line to the initial linear part of the f/I curve. e. Comparison of the minimum current required to evoke APs (rheobase) in “responders” (n=9) and “non-responders” (n=6). f,g. Comparison of input resistance (f) and resting membrane potential (g) in “responders” (n=15) and “non-responders” (n=6). Non-significant comparisons denoted with “n.s” representing $p > 0.05$ using non-parametric Wilcoxon-Mann-Whitney two sample rank test. Pooled data represents mean ± SEM. Source data are provided as a Source Data file.

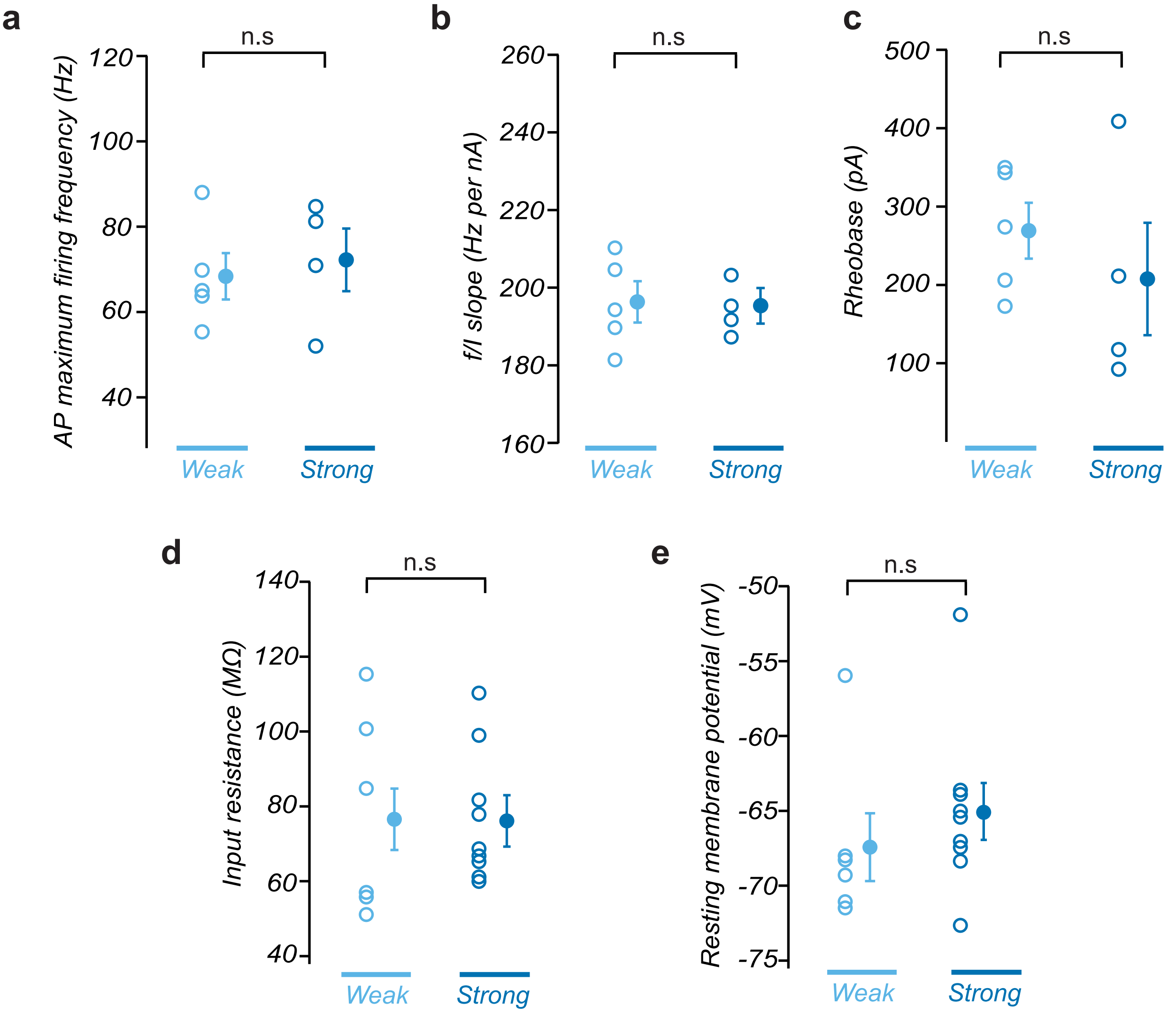

Supplementary Fig. 6: POm neurons receiving weak and strong SC input have similar active and passive properties. a. Comparison of AP firing frequency in POm neurons receiving weak (light blue; n=5) and strong (dark blue; n=4) SC input. b. Comparison of f/I slope in POm neurons receiving weak (n=5) and strong (n=4) SC input. The slope was calculated by fitting a line to the initial linear part of the f/I curve. c. Comparison of the minimum current required to evoke APs (rheobase) in POm neurons receiving weak (n=5) and strong (n=4) SC input. d,e. Comparison of input resistance (d) and resting membrane potential (e) in POm neurons receiving weak (n=9) and strong (n=9) SC input. Non-significant comparisons denoted with "n.s" representing $p > 0.05$ using non-parametric Wilcoxon-Mann-Whitney two sample rank test. Pooled data represents mean ± SEM. Source data are provided as a Source Data file.
